# Intraoperative cultures during appendectomy in children are poor predictors of pathogens and resistance patterns in cultures from postoperative abscesses

**DOI:** 10.1007/s00383-018-04428-3

**Published:** 2019-01-08

**Authors:** Martin Dahlberg, Markus Almström, Tomas Wester, Jan F. Svensson

**Affiliations:** 1 Department of Clinical Science and Education, Department of Surgery, Södersjukhuset, Karolinska Institutet, Stockholm, Sweden; 2 Department of Pediatric Surgery, Astrid Lindgren Children’s Hospital, Karolinska University Hospital, Stockholm, Sweden; 3 Department of Women’s and Child’s Health, Karolinska Institutet, Stockholm, Sweden; 4 Department of Surgery, Stockholm South General Hospital (Södersjukhuset), Sjukhusbacken 10, 118 83 Stockholm, Sweden

**Keywords:** Complicated appendicitis, Antibiotic coverage, Microbial content

## Abstract

**Background:**

Intraoperative cultures are commonly sent in complicated appendicitis. Culture-guided antibiotics used to prevent postoperative infectious complications are debated. In this study, we describe the microbial overlap between intraoperative and abscess cultures, and antibiotic resistance patterns.

**Method:**

A local register of a children’s hospital treating children 0–15 years old with appendicitis between 2006 and 2013 was used to find cases with intraoperative cultures, and cultures from drained or aspirated postoperative intraabdominal abscesses. Culture results, administered antibiotics, their nominal coverage of the identified microorganisms, and rationales given for changes in antibiotic regimens were collected from electronic medical records.

**Results:**

In 25 of 35 patients who met inclusion criteria, there was no overlap between the intraoperative and abscess cultures. In 33 of 35 patients, all identified intraoperative organisms were covered with postoperative antibiotics. In 14 patients, organisms in the abscess culture were not covered by administered antibiotics. Enterococci not found in the intraoperative culture were found in 12 of 35 abscesses. We found no difference in the antibiotic coverage between rationales given for antibiotic changes.

**Conclusion:**

The overlap between intraoperative cultures and cultures from subsequent abscesses was small. Lack of antibiotic coverage of intraoperative cultures was not an important factor in abscess formation.

**Electronic supplementary material:**

The online version of this article (10.1007/s00383-018-04428-3) contains supplementary material, which is available to authorized users.

## Introduction

In patients with perforated appendicitis, surgery and antibiotics are used to limit bacterial contamination. Commonly, broad-spectrum antibiotic regimens are used to cover the most common fecal bacterial pathogens that are released into the peritoneal cavity once the appendix perforates. Findings of the same bacteria in distant abscesses and periappendiceal abscesses were described in patients with appendicitis by Altemeier in the 1930s [1], but since then antibiotic treatment regimens have changed dramatically.

Two main philosophies can be identified in the antibiotic regimens used to cover organisms that form intraabdominal abscesses (IAA) after complicated appendicitis: (1) to cover the most common pathogens empirically and change antibiotics based on clinical deterioration, or according to cultures from the abscess if an IAA forms, and (2) to start empiric treatment and send cultures from the primary operation and change antibiotics to cover organisms found in the cultures. This intraoperative swabbing of the peritoneum to identify resistant organisms is controversial [2–5], and was reviewed by Davies et al. [6]. It is not certain that the dominating organisms found initially are those that establish abscesses, and several smaller series indeed show a large discrepancy between intraoperative and IAA cultures [7–10], and some data suggests that guiding antibiotics by peritoneal swabs gets even worse results than empiric broad-spectrum antibiotics [9].

To address the population where antibiotic choice and treatment duration had not successfully prevented infectious complications, we examined pediatric patients operated for appendicitis where subsequent IAAs were drained or aspirated and sent for cultures. By comparing intraoperative cultures and IAA cultures, the aim was to describe to what extent the organisms overlap and if lack of initial antibiotic coverage could be identified.

## Methods

A local register for all children, aged 0–15 years, undergoing appendectomy during 2006–2013 was used to identify those who had a postoperative IAA. Children in whom (1) an intraoperative culture was obtained, and (2) in whom the IAA was drained (percutaneously for aspirate or drainage, or with open drainage) and material sent for cultures were included in this study. We compared the intraoperative culture with the IAA culture with respect to organisms present and their resistance patterns. Antibiotic regimens in the immediate postoperative phase, any changes in the antibiotic regimen, as well as any documented rationale for changing antibiotics, e.g., guided by cultures or empirical broadening due to clinical deterioration were recorded. When both empiric and guided approaches were used, we classified the case as guided by cultures. Histopathology reports were examined in all patients to classify perforated, non-perforated gangrenous, or non-perforated phlegmonous appendicitis [11]. In some cases, the operative notes were needed to classify the degree of appendicitis. Data on the length of hospital stay were retrieved from the local register.

We classified organisms obtained in the intraoperative and IAA cultures, respectively, according to their susceptibility to antibiotics given from the primary operation to the time of IAA drainage and culture. Where susceptibility was not explicitly stated for a given antibiotic, we assumed that *Streptococcus anginosus* and alpha-streptococci were covered by meropenem, piperacillin/tazobactam, and cephalosporins if sensitive to penicillin, and that *B. fragilis* was sensitive to meropenem. *Enterococcus faecium* not explicitly sensitive to meropenem (including strains sensitive to imipenem) was assumed not sensitive to meropenem. When antibiotic susceptibility was unknown, this was recorded as unknown.

The Mann–Whitney *U* and Kruskal–Wallis tests were used to test the differences in hospital stay, and Fisher’s exact test for pairwise comparisons in two-way tables and in 3 × 3 tables with significance level set at *p* = 0.05 and with a two-tailed hypothesis. Analyses were performed with R (version 3.2.4, R Foundation for Statistical Computing, Vienna, Austria).

The study was approved by the regional Ethics Review Board in Stockholm (ref no 2014/1018-31/4).

## Results

### Study population

**Fig. 1:**
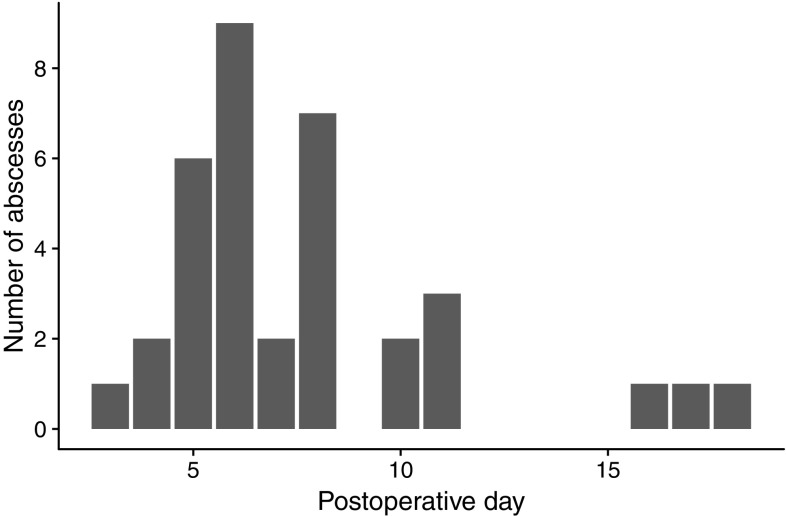
Distribution of postoperative days to IAA drainage

Between 2006 and 2013, a total of 2888 patients underwent appendectomy for acute appendicitis at Karolinska University Hospital, Stockholm, Sweden. In 2216 patients (77%), a culture was taken at the time of appendectomy. Perforated appendicitis was cultured intraoperatively in 94% of cases, whereas 72% were cultured in non-perforated appendicitis. One-hundred and twenty patients (4.2%) were diagnosed with a postoperative abscess, and 42 patients (1.5%) had an intervention targeting the abscess. Thirty-five patients met the inclusion criteria for this study. The median age was 11.8 years (IQR 9.3–13.5). Fifteen patients (43%) were female. Twenty-eight patients had a laparoscopic appendectomy (two conversions to open surgery) and seven had open appendectomies. Perforated appendicitis was found in 30 out of 35 included patients. Two patients had phlegmonous appendicitis, and three gangrenous appendicitis, one in whom the appendix perforated intraoperatively. IAAs were diagnosed and drained or aspirated, with material sent for cultures, on median postoperative day six (range 3–18, see Fig. 1).

Between 2006 and 2013, the locally recommended postoperative antibiotic regimens for complicated appendicitis changed from cefotaxime/metronidazole, to trimethoprim (TMP)/sulfamethoxazole(SMX)/metronidazole, to meropenem/metronidazole, which was reflected in this cohort of patients (Supplementary Table 1).

### Culture findings

In Table 1, the organisms found in the intraoperative and IAA cultures are tabulated. *B. fragilis, E. coli*, and *S. anginosus* were the most common organisms found intraoperatively, whereas various *Enterococci, S. anginosus*, and no growth of bacteria were the most common findings in abscesses.

**Table 1:** Distribution of organisms in intraoperative and IAA cultures

Intraoperative organism	Present in *N* cultures	IAA organism	Present in *N* cultures
*B. fragilis*	26	No growth	11
*E. coli*	21	*E. faecium*	6
*S. anginosus*	18	*S. anginosus*	6
*Mixed anaerobes*	8	*E. coli*	5
*P. aeruginosa*	6	*B. fragilis*	3
*Eikenella corrodens*	3	*C. albicans*	3
*ESBL E. coli*	3	*E. faecalis*	3
*E. faecium*	2	*Mixed anaerobes*	2
*Pseudomonas sp*	2	*Enterococcus avium*	2
No growth, *Alpha streptococcus, Enterobacter aerogenes, H. influenzae, S. aureus, Alpha-hemolytic streptococcus*	1	*Enterococcus species*	2
		*CoNS*	2
		*P. aeruginosa*	2
		*Eikenella corrodens, Enterococcus casseliflavus, ESBL E. coli, Fusobacterium spp, Hafnia alvei, Prevotella sp, Propionibacterium, Saccharomyces cerevisiae, Alpha-hemolytic streptococcus*	1

*CoNS* coagulase-negative staphylococci, *ESBL* extended spectrum beta-lactamase

Overlap in at least one organism between the intraoperative and IAA cultures was found in 10 out of 35 patients (Supplementary Figure A1). The overlapping organisms were covered by antibiotics in eight of ten cases according to the reported sensitivities. Organisms found in the intraoperative culture were covered by postoperative antibiotics, including changes to second- and third-line antibiotics, in the majority of cases (Table 2), but IAA organisms were fairly evenly divided between covered, uncovered, and no growth identified.

**Table 2:** Antibiotic coverage of intraoperative and IAA cultures, with rationale for any postoperative antibiotic changes

	Empiric	Guided	No change	All
Intraoperative culture^#^				
Covered, *n*	8	13	11	32
Not covered, *n*	0	0	2	2
No growth, *n*	0	0	1	1
IAA culture^§^				
Covered, *n*	5	2	2	9
Not covered, *n*	1	8	5	14
No growth, *n*	2	3	7	12
Hospital stay**	11.1 (0.77–24)	13.5 (8.6–24)	7.8 (1.6–13)*	10.7

**In days, median (range)

*p < 0.001 No change vs guided (Mann–Whitney *U* test), Kruskal–Wallis rank test for all *p* = 0.002

^#^*p* = 0.348, Fisher’s exact test

^§^*p* = 0.0618, Fisher’s exact test

### Empiric vs guided changes in antibiotics

To further understand how antibiotics were used and how this affected antibiotic coverage, subgroups were defined based on the documented rationale for changes in the antibiotic regime (Table 2). No pairwise significant differences were found between groups. To estimate the burden of disease in the different approaches to antibiotic use, we compared the distributions of hospital stay in the subgroups and found that the difference between guided antibiotics and no change was significant (Table 2). The median hospital stay was 10.7 days, indicating the severity of disease in this population. To ensure that the new antibiotic was relevant with respect to the IAA, i.e., had time to penetrate the abscess, the time from antibiotic change to drainage was investigated. Empiric changes were done in median 2.5 days (2 doses minimum, 15 days maximum) prior to drainage. In patients with intraoperative culture-guided changes, these were made in median 3 days prior to the IAA drainage, with a maximum of 8 days prior, and for 2 patients out of 13, the antibiotic was changed 2 and 3 days, respectively, after drainage.

### Important subgroups

One important group to consider is patients where IAA organisms were covered by antibiotics according to the resistance pattern, but appeared in the abscess anyway. Out of the nine such cases, four had *S. anginosus*, three had *E. coli* (one isolate producing extended spectrum beta-lactamase [ESBL]), and two patients with *B. fragilis* or *C. albicans* were identified.

Enterococci not found in the intraoperative culture were found in 12 of 35 IAA cultures and were, as a group, the most common organisms in IAAs not found in intraoperative cultures. *C. albicans* was found in IAA cultures in three patients (Supplementary Fig. A1: #2, #5, #7), and *Saccharomyces cerevisiae* was found in one patient (Supplementary Fig. A1: #11). Only in one patient with *C. albicans* an antifungal was used, reflecting that not all organisms were considered clinically relevant.

*S. anginosus* was found in 18 (51%) and 6 (17%) of intraoperative and IAA cultures, respectively. Out of the six patients with *S. anginosus* in the IAA, five also had *S. anginosus* in the intraoperative culture. Cultures with ESBL *E. coli* were found intraoperatively in three patients, and in one of these (Supplementary Fig. A1: #30) ESBL *E. coli* was also found in the abscess. The latter strain was susceptible in vitro to meropenem which was the antibiotic given before draining the IAA.

### Changed antibiotic susceptibility within patients

Changes in antibiotic susceptibility between intraoperative and IAA cultures were only found in four patients. In one patient (Supplementary Fig. A1: #27, TMP/SMX/metronidazole postoperatively), the *S. anginosus* strain found intraoperatively was sensitive to TMP/SMX, but resistant to TMP/SMX in the IAA. In the second patient (Supplementary Fig. A1: #22, meropenem/metronidazole postoperatively), *E. coli* was found resistant to TMP/SMX, but a sensitive *E. coli* was found in the IAA. The third patient (Supplementary Fig. A1: #17, cefotaxime/metronidazole and meropenem postoperatively) had more intricate changes, with *S. anginosus* and *B. fragilis* found sensitive to clindamycin, but resistant in the IAA. The same patient had *B. fragilis* initially intermediately sensitive to piperacillin/tazobactam, but fully sensitive in the IAA. The fourth patient (Supplementary Fig. A1: #21, TMP/SMX/metronidazole postoperatively) had intraoperative *B. fragilis* sensitive to piperacillin/tazobactam, and intermediately sensitive *B. fragilis* in the IAA. Overall, these changes in susceptibility did not cause any difficulty in covering the respective organisms with the postoperative antibiotic regimens used, and in only one case possible resistance to a given antibiotic developed.

## Discussion

We found that the overlap between intraoperative cultures and IAA cultures was limited, with only 29% (10/35) of patients having some overlap in cultured organisms. Although 91% (32/35) of intraoperative cultures were covered by given antibiotics, only 40% (14/35) of patients had IAA organisms resistant to given antibiotics. The main mode of abscess development seems to be from resistant organisms not dominant in the intraoperative culture, typically *Enterococci*, or by organisms in vitro sensitive to the antibiotics administered resulting in no growth in cultures from the IAA. Changing antibiotic susceptibility between perforation and abscess formation was not found to be an important factor for abscess formation.

The group without changes in the postoperative antibiotics probably had a clinically less severe postoperative course, as changes in the antibiotics were not attempted, and is corroborated by the shorter time in hospital in this group. It can also be speculated that empiric alterations in the antibiotic regimen were motivated by clinical deterioration and could constitute a sicker group. However, this is not supported by the data, where instead the patients with guided changes appeared to have stayed longer in the hospital, although the difference was not statistically significant.

Two patterns that we noted regarding the most common organisms in IAAs were that *S. anginosus* tended to be present also in intraoperative culture (5/6), whereas *Enterococci* were found de novo in the abscesses. When *S. anginosus* is present in the intraoperative swab, previous studies indicate that abscess formation is overrepresented [8, 12, 13], and that *S. anginosus* is overrepresented in complicated appendicitis [14]. We believe that organisms found in the IAA are present at the time of initial surgery, unless some unknown mechanism of contamination other than the perforation event itself occurs in a large proportion of the patients. The inability to identify these organisms in intraoperative cultures is probably due to other species being more predominant in the cultures. PCR-based methods could possibly help in identifying a broader range of intraoperative organisms, but is of unknown clinical value in this setting. The large subgroup of patients with *Enterococci* in the IAA are worrying in that they are not necessarily covered by initial antibiotic regimens, a natural resistance to many common antibiotics, and because of an increase in vancomycin-resistant *Enterococci* (VRE). Our current antibiotic regimen in complicated appendicitis has now changed again to include piperacillin/tazobactam, with better coverage of, at least, *E. faecalis*.

There is some data to suggest that not covering the organisms found at the time of surgery is associated with increase in superficial wound infections and a non-significant increase in abscess formation [15] and some authors recommend cultures to guide antibiotic strategy in complex cases [16]. However, recent work on treatment duration suggests shortening antibiotic regimens to 3–5 days [17, 18], where intraoperative culture results are not always ready, and antibiotics therapies guided by those cultures would prolong treatment to beyond 5 days [19]. If antibiotic treatment length is not the main factor for improvement beyond 5 days, the distribution of hospital stay and time to IAA drainage in this study suggests that a more active approach to draining abscesses could have improved outcomes in terms of hospital stay and minimized exposure to antibiotics.

An additional obstacle in reviewing the effect of peritoneal swabs is that the pathogens causing postoperative IAAs are not characterized unless abscesses are found and drained or aspirated, and that intraoperative antibiotics are usually already administered reducing the sensitivity of the culture. There is also no clear consensus on where the most representative culture should be sampled in the contaminated abdomen to best predict how to prevent abscess formation.

A bias in this retrospective review of patients who developed IAAs is that we cannot compare how many IAAs were prevented by changes in antibiotics due to information from the intraoperative culture. Looking instead at all patients who underwent treatment for perforated appendicitis, previous work found increased infectious complications, longer fever duration, and longer hospitalization with tailored antibiotic changes compared with empiric changes [9]. Again, retrospective designs cannot determine which approach is best applied prospectively, but matching disease severity could help reduce bias. Randomized studies using the two philosophies outlined above are not available, but requires large numbers of patients because abscess formation is relatively rare, 13–19% in the pediatric subgroup with perforated appendicitis [20–23], and the predicted differences between groups is probably not large as broad-spectrum antibiotics are used initially in both groups. A further weakness to the retrospective medical record review is that the rationale for altering antibiotics or how and when the cultures were used is not necessarily explicitly stated, and likely varied between surgeons and possibly between patient characteristics like age and symptoms. Because of this heterogeneity and the limited number of patients in this study, subgroup analyses should only be considered hypothesis generating. One limitation in the microbiology reports on antibiotic susceptibilities is that not all common drugs used in the study patients were included explicitly. Most such situations, like carbapenem susceptibility in *S. anginosus*, could be reasonably settled with the clinically based assumptions described here. A further limitation is that only 77% of patients were cultured intraoperatively. In the subgroup at highest risk for IAA, i.e., those with perforated appendicitis, 94% were cultured. We find non-adherence to the local recommendation of culturing all appendicitis cases to be an unlikely source of serious bias given the dominance of perforated appendicitis cases in this IAA cohort. There was considerable variation in the antibiotics given over this 8-year period and this may make the interpretation of the results more difficult. However, local recommendations were followed in the postoperative antibiotic given in most cases, and the strategy with respect to sending intraoperative cultures and letting them guide antibiotic changes to be largely divorced from the specifics of the antibiotic type as long as appropriate initial broad-spectrum antibiotics are used.

Because antibiotic choice changes over time as resistance patterns change, it is important to find practice guidelines that help not only with what antibiotic to use, but with the rationale for changing and stopping antibiotics. Clearly, as we show, intraoperative cultures are not easy to interpret as they poorly reflect the contents of IAAs, and to narrow treatment to intraoperative findings could be misleading given the frequent occurrence of other organisms in IAAs. Empiric broad-spectrum coverage of the common pathogens is reasonable going forward, and in the light of this study and previous work the value of intraoperative cultures is doubtful.

## Electronic supplementary material

Below is the link to the electronic supplementary material.

Supplementary material 1 (DOCX 88 KB)

**Supplementary Fig. A1**: Antibiograms with organisms and antibiotic susceptibilities for the 35 patients with intraoperative (•, ◯ respectively) cultures (EPS 1772 KB)
