# Supplementary material for: Intraoperative cultures during appendectomy in children are poor predictors of pathogens and resistance patterns in cultures from postoperative abscesses
Source: Pediatr Surg Int. 2019 Jan 8;35(3):341–6. doi: 10.1007/s00383-018-04428-3 (PMC6394779; doi:10.1007/s00383-018-04428-3)
Supplement: Supplementary file 1 — Supplementary material 1 (DOCX 88 KB) [file 383_2018_4428_MOESM1_ESM.docx]

**Supplementary Table 1** Antibiotic regimens (number of patients in parentheses).

| Initial antibiotics | Second and third line antibiotics |
| --- | --- |
| Cefotaxime + Metronidazole (16) | No change (5)  Imipenem/Cilastatin (2) + Fluconazole (1)  Meropenem (2) + Cloxacillin (1)  Piperacillin/Tazobactam (1) + Metronidazole (1)  Ceftazidime (1)  Ciprofloxacin + Metronidazole (1)  TMP/SMX + Metronidazole (1) |
| Meropenem + Metronidazole (12) | No change (7)  TMP/SMX + Metronidazole (2)  + Ampicillin (1)  Piperacillin/Tazobactam + Metronidazole (1)  TMP/SMX + Metronidazole changed to Ciprofloxacin + Metronidazole (1) |
| TMP/SMX + Metronidazole (5) | No change (2)  Meropenem + Metronidazole (2)  Piperacillin/Tazobactam + Metronidazole (1) |
| Piperacillin/Tazobactam + Metronidazole (1) | Meropenem + Ciprofloxacin + Metronidazole (1) |
| Meropenem (1) | + Metronidazole + Ampicillin(1) |

**Supplementary Fig. A1**: Antibiograms with organisms and antibiotic susceptibilities for the 35 patients with intraoperative (•) and IAA (🌣) cultures
